# The emergence of multidrug-resistant clone ST664 *Pseudomonas aeruginosa* in a referral burn hospital, Isfahan, Iran

**DOI:** 10.1186/s41038-017-0092-x

**Published:** 2017-10-10

**Authors:** Hamid Vaez, Hajieh Ghasemian Safaei, Jamshid Faghri

**Affiliations:** 1 Department of Microbiology, School of Medicine, Zabol University of Medical Sciences, Zabol, Iran; 2 Department of Microbiology, School of Medicine, Isfahan University of Medical Sciences, Isfahan, Iran

Dear editor,

Treatment of infection caused by multidrug-resistant (MDR) *Pseudomonas aeruginosa* (*P. aeruginosa*) strains due to simultaneously resistance to different class of antibiotics is a major burn unit concern worldwide [1]. Multidrug-resistant isolates of *P. aeruginosa* (MDR-*P. aeruginosa*) are non-susceptible to at least three antibiotics belonged to different classes especially carbapenems, fluoroquinolones, and aminoglycosides [2]. Since determination of clonal relatedness among bacterial infectious agents is an essential pre-requisite to take appropriate infection control practices, the aim of this study was to investigate the genetic diversity and document the presence of high-risk clone of MDR-*P. aeruginosa* in burn wound infections.

A cross-sectional study was carried out between March 2013 and September 2013 at a referral burn hospital of Isfahan province, Iran. The hospital is the only burn referral center in the region, serving about five million people. In total, 100 burn patients admitted to Intensive Care Unit (ICU) during period of study were included. During the study period, sterile swabs were used for sample collection from wounds of burn patients whom, based on clinical signs, we suspected had a wound infection. After identification, antibiotic resistance profile was determined by disk diffusion method. Finally, MDR-clone of identified *P. aeruginosa* was subjected to multi locus sequence typing (MLST), according to previously described procedures [3].

The specimens of 30 (30%) patients yielded bacterial growth. From these, 13 (43.3%) patients were female. The susceptibility patterns of isolated bacteria against applied antibiotics were as follows: ceftazidime (16.7%; 5/30), polymyxin B (100%; 30/30), gentamicin (73.3%; 22/30), piperacillin (6.7%; 2/30), ceftriaxone (6.7% 2/30), imipenem (73.3%; 22/30), ciprofloxacin (50%; 15/30), aztreonam (13.3%; 4/30), meropenem (70%; 21/30), and amikacin (73.3%; 22/30). Out of the 30 isolated strains of *P. aeruginosa*, 8 (26.7%) were MDR, showing resistance against all applied antibiotics except for polymyxin B. We found four different sequence types (STs); ST235, ST233, ST357, and ST664.

In present study, *P. aeruginosa* isolates were mostly resistant against piperacillin (93.3%), ceftriaxone (93.3%), and aztreonam (86.7%). These findings are similar to other studies performed in Iran [4, 5]. Salimi et al. reported that many isolates of *P. aeruginosa* collected from burn patients referred to Mottahari hospital in Tehran province were resistant to used antibiotics, ceftizoxime (87%), aztreonam (80.2%), kanamycin (79.4%), and ceftazidime (74.8%) [6]. MLST of *P. aeruginosa* revealed that some MLST STs such as ST235, ST175, and ST111 are high risk and epidemic clones because these STs showed resistance against multiple different classes of antibiotics, also were associated with high mortality and morbidity rate worldwide [7, 8]. Four different STs in MDR-*P. aeruginosa* were detected (Table 1). The most common type was ST664 and ST357, which were detected in four and two isolates, respectively. Other types including ST233 and ST235 were also detected in one isolate. The majority of our STs including ST233, ST664, and ST357 were identified for the first time in Isfahan. Study conducted by Fazeli et al. in Isfahan province showed that the majority of MDR-clone of *P. aeruginosa* belonged to ST235 [9]. Likewise, results of different independent studies revealed that ST235 is an international clone and distributed worldwide [7, 8]. For example, Cholley et al. reported that ST235 was the most common MDR-clone isolated from France hospitals [8]. In another study performed in different hospitals of Korea, ST235 was known to be the most frequent type with higher rate of non-susceptibility to different classes of antibiotics including penicillins, expanded-spectrum cephalosporins, carbapenems, aminoglycosides, and fluoroquinolones [10].

**Table 1:** Characteristics of MDR-*P. aeruginosa* isolated from burn wound infection

Isolates	Allelic profiles (*acsA, aroE guaA, mutL, nuoD, ppsA, trpE*)	ST	Antibiotic resistance pattern
1	2-4-5-3-1-6-11	357	R: CAZ, IMP, MEM, CIP, ATM, PIP, AMK, GM
2	2-4-5-3-1-6-11	357	R: CAZ, IMP, MEM, CIP, ATM, PIP, AMK, GM
3	38-11-3-13-1-2-4	235	R: CAZ, IMP, MEM, CIP, ATM, PIP, AMK, GM
4	9-5-11-3-4-40-18	664	R: CAZ, IMP, MEM, CIP, ATM, PIP, AMK, GM
5	9-5-11-3-4-40-18	664	R: CAZ, IMP, MEM, CIP, ATM, PIP, AMK, GM
6	9-5-11-3-4-40-18	664	R: CAZ, IMP, MEM, CIP, ATM, PIP, AMK, GM
7	9-5-11-3-4-40-18	664	R: CAZ, IMP, MEM, CIP, ATM, PIP, AMK, GM
8	16-5-30-11-4-31-41	233	R: CAZ, IMP, MEM, CIP, ATM, PIP, AMK, GM

*MDR-P. aeruginosa* Multidrug-resistant isolates of *P. aeruginosa*, *ST* sequence type, *R* resistance, *CAZ* ceftazidime, *IMP* imipenem, *MEM* meropenem, *CIP* ciprofloxacin, *ATM* aztreonam, *PIP* piperacillin, *AMK* amikacin, *GM* gentamicin
